# ^18^F-fluorodeoxyglucose positron-emission tomography (FDG-PET)-Radiomics of metastatic lymph nodes and primary tumor in non-small cell lung cancer (NSCLC) – A prospective externally validated study

**DOI:** 10.1371/journal.pone.0192859

**Published:** 2018-03-01

**Authors:** Sara Carvalho, Ralph T. H. Leijenaar, Esther G. C. Troost, Janna E. van Timmeren, Cary Oberije, Wouter van Elmpt, Lioe-Fee de Geus-Oei, Johan Bussink, Philippe Lambin

**Affiliations:** 1 Department of Radiation Oncology (MAASTRO), GROW–School for Oncology and Developmental Biology, Maastricht University Medical Center (MUMC +), Maastricht, the Netherlands; 2 Institute of Radiooncology—OncoRay, Helmholtz-Zentrum Dresden-Rossendorf, Dresden, Germany; 3 Department of Radiotherapy and Radiation Oncology, Medical Faculty and University Hospital Carl Gustav Carus of Technische Universität Dresden, Dresden, Germany; 4 OncoRay, National Centre for Radiation Research in Oncology, Medical Faculty and University Hospital Carl Gustav Carus of Technische Universität Dresden, Helmholtz-Zentrum Dresden-Rossendorf, Dresden, Germany; 5 Department of Radiology and Nuclear Medicine, Radboud UMC, Nijmegen, the Netherlands; 6 Department of Radiology, Leiden University Medical Center, Leiden, the Netherlands; 7 Biomedical Photonic Imaging Group, MIRA Institute, University of Twente, Enschede, the Netherlands; 8 Department of Radiation Oncology, Radboud University Medical Center, Nijmegen, the Netherlands; University of South Alabama Mitchell Cancer Institute, UNITED STATES

## Abstract

**Background:**

Lymph node stage prior to treatment is strongly related to disease progression and poor prognosis in non-small cell lung cancer (NSCLC). However, few studies have investigated metabolic imaging features derived from pre-radiotherapy ^18^F-fluorodeoxyglucose (FDG) positron-emission tomography (PET) of metastatic hilar/mediastinal lymph nodes (LNs). We hypothesized that these would provide complementary prognostic information to FDG-PET descriptors to only the primary tumor (tumor).

**Methods:**

Two independent cohorts of 262 and 50 node-positive NSCLC patients were used for model development and validation. Image features (i.e. Radiomics) including shape and size, first order statistics, texture, and intensity-volume histograms (IVH) (http://www.radiomics.io/) were evaluated by univariable Cox regression on the development cohort. Prognostic modeling was conducted with a 10-fold cross-validated least absolute shrinkage and selection operator (LASSO), automatically selecting amongst FDG-PET-Radiomics descriptors from (1) tumor, (2) LNs or (3) both structures. Performance was assessed with the concordance-index. Development data are publicly available at www.cancerdata.org and Dryad (doi:10.5061/dryad.752153b).

**Results:**

Common SUV descriptors (maximum, peak, and mean) were significantly related to overall survival when extracted from LNs, as were LN volume and tumor load (summed tumor and LNs’ volumes), though this was not true for either SUV metrics or tumor’s volume. Feature selection exclusively from imaging information based on FDG-PET-Radiomics, exhibited performances of (1) 0.53 –external 0.54, when derived from the tumor, (2) 0.62 –external 0.56 from LNs, and (3) 0.62 –external 0.59 from both structures, including at least one feature from each sub-category, except IVH.

**Conclusion:**

Combining imaging information based on FDG-PET-Radiomics features from tumors and LNs is desirable to achieve a higher prognostic discriminative power for NSCLC.

## Introduction

Non-small cell lung cancer (NSCLC) patients often present with hilar and/or mediastinal lymph node involvement at diagnosis or during the course of disease. Lymph node stage prior to treatment is strongly related to disease progression and worse prognosis [1]. Furthermore, it affects treatment selection and target volume definition, for metastatic lymph nodes in patients eligible for high-dose (chemo)radiotherapy [2].

In this study we hypothesized that the local selection of more aggressive cancer cells in the metastatic hilar/mediastinal lymph nodes, being likely to determine prognosis, may provide an additional and valuable source of information to the primary tumor for NSCLC patients. A Radiomics-based approach comprises the extraction of a large set of imaging descriptors [3]. The underlying hypothesis is that biomarkers of imaging phenotypes deliver complementary and clinically relevant information, which could be incorporated into individualized radiation oncology approaches and shared decision-making tools [4–7]. To demonstrate this, we performed a combined Positron Emission Tomography (PET) Radiomics analysis of metabolic activity as measured with ^18^F–fluorodeoxyglucose (FDG) uptake in both primary tumor and metastatic lymph nodes, and further validated these results in an independent cohort.

## Patients and methods

### Development cohort

#### Patient population

The prospective data collection was approved by the Institutional Review Board of the Department of Radiotherapy of Maastricht University Medical Center (MAASTRO clinic) (clinicaltrials.gov NCT00522639). Electronic medical charts of NSCLC patients were reviewed. Patients undergoing surgery, Stereotactic Body Radiotherapy (SBRT) or palliative treatment, or who had a previous malignancy within five years prior to diagnosis were excluded from analysis. A total of 343 NSCLC patients (stage I-IIIB) referred to curative treatment (between May 2006 and September 2012) were selected for the development cohort. Out of these, 262 patients (76%) had metastatic lymph nodes. Patients received high-dose radiotherapy (RT), planned on a dedicated FDG-PET-CT scan, combined with chemotherapy. Clinical follow-up was performed according to national guidelines. All patients in the development cohort provided informed written or verbal consent to data inclusion in clinical studies.

#### Image acquisition

Before scanning, patients fasted for at least 6 hours. Two different protocols were used: until December 2010, the total dose of FDG was calculated as (bodyweight x 4 + 20) MBq, and as from January 2011, the administered dose was (2.5 x bodyweight) MBq as defined by the NEDPAS protocol [8]. FDG-PET-CT images were taken 60-minutes post injection. Data acquired until December 2006 were gathered on Siemens Biograph 16 CT-PET scanner, and from that time onwards on a Siemens Truepoint 40 CT-PET (Siemens Healthcare AG, Erlangen, Germany). An Ordered Subset Expectation Maximization 2D 4 iterations 8 subsets (OSEM2D 4i8s) algorithm was used for image reconstruction using post-reconstruction 5mm Gaussian filtering, and voxel size of 4.0728 x 4.0728 x 3 (mm). Model-based methods were applied for scatter correction. All PET scans were corrected for attenuation using the mid-ventilation phase of the 4DCT or a 3DCT thorax in case the 4DCT was not of sufficient image quality due to irregular breathing of the patient. All exams were corrected for random events and decay.

### Validation cohort

#### Patient population

The validation cohort included 215 stage I-IIIB NSCLC patients, treated with primary radio(chemo)therapy between May 2006 and October 2012 at the Department of Radiotherapy of Radboud UMC Nijmegen, following same inclusion and exclusion criteria as for development cohort. In total 115 (53%) patients were node-positive, of which 50 (23%) had an available treatment planning FDG-PET-CT. All patients in the validation cohort provided informed written or verbal consent to data inclusion in clinical studies.

#### Image acquisition

Before scanning, patients fasted for at least 6 hours. FDG-PET scans were performed 60 minutes after intravenous injection of approximately 250 MBq FDG (Covidien, Petten, the Netherlands) and 10 mg furosemide. PET scans were performed on Siemens Biograph Duo (Siemens Medical Solutions USA, Inc.) using three-dimensional emissions of 4 minutes per bed position as described previously [9]. A low-dose CT scan for localization and attenuation-correction purposes was acquired. Scanning parameters included 40 mA·s (50 mA·s for patient weight >100 kg and 60 mA·s if >120 kg), 130 kV, 5-mm slice collimation, 0.8-second rotation time, and pitch of 1.5, reconstructed with 3-mm slices for smooth coronal representation. An OSEM2D 4i16s algorithm was used for PET image reconstruction, with a voxel size of 5.3 x 5.3 x 3.375 (mm). All PET scans were corrected for attenuation using CT and simulation approaches. Model-based methods were applied for scatter correction. All exams were corrected for random events and decay.

### Regions of interest (ROI)

Images were imported into the research treatment planning system Xio/Focal (development cohort) and Eclipse (validation cohort) using the DICOM protocol. The primary gross tumor volume (tumor) and metastatic hilar/mediastinal lymph nodes (LN), identified as PET positive and/or proven by endoscopic ultrasound bronchoscopy/esophagoscopy (EBUS/EUS), were manually delineated by experienced radiation oncologists on the fused FDG-PET-CT images, and used as the regions of interest for analysis [10–12]. A single structure representing all metastatic lymph nodes, regardless of their number, was derived for each patient (see online appendix for further details).

### Image analysis

In-house developed software was used to extract Radiomics descriptors from the FDG-PET scans [13–16]. Imaging descriptors comprised first order statistics (n = 16), shape and size (n = 13), intensity volume histograms (n = 45), and textural features describing the spatial distribution of voxel intensities (n = 44). Textural features were calculated from grey-level co-occurrence (GLCM), grey-level run-length (GLRLM) and grey-level size-zone texture matrices (GLSZM). To determine these matrices, images were first discretized with a bin width of 0.5 (Standardized Uptake Value or SUV), according to:

$$I_{D}(x)=\left\lceil \frac{I(x)}{0,5}\right\rceil -\min\left(\left\lceil \frac{I(x)}{0,5}\right\rceil \right)+1$$

Where *I* is the original image, *I(x)* represents the SUV of voxel *x*, and *I*_*D*_ is the resulting discretized image [15]. Texture matrices were then constructed by considering 26 connected voxels (i.e. voxels were considered to be neighbors in all 13 directions in three dimensions) at a distance of 1 voxel. Features derived from GLCM and GLRLM were calculated by averaging their value over all 13 directions. Forty-four textural features were extracted (22 GLCM, 11 GLRLM and 11 GLSZM). In total, 118 imaging features were calculated based on the FDG-PET distribution within ROI, which mathematical formulations are detailed in the work of Leijenaar et al. [14]. Image analysis was performed in Matlab R2012b (The Mathworks, Natick, MA), based on an adapted version of Computational Environment for Radiotherapy Research (CERR) [17].

### Statistical analysis

#### Study parameters/endpoints

Primary endpoint of the study was overall survival (OS), defined as the time from the start of radiotherapy until the last day of follow-up or death due to any cause, and was available for all patients under analysis. A patient still alive at the end of the study was regarded as right-censored.

#### Univariable analysis

A pre-feature selection was performed as detailed in the online appendix. Imaging and clinical features were analyzed as continuous variables in a univariable Cox regression. In addition, a correlation analysis was conducted for maximum, peak, and mean SUV, and volume of tumor and LNs, and tumor load (sum of tumor and LN volumes).

#### Multivariable analysis

**Fig 1:**
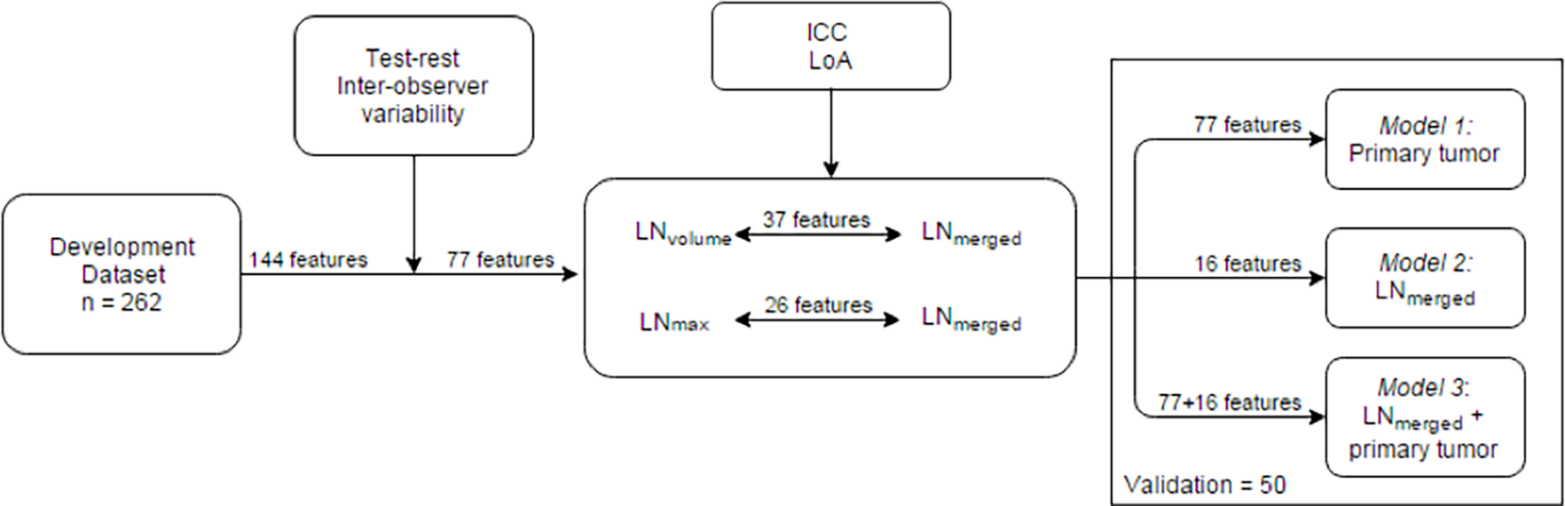
Diagram of the workflow followed in the multivariable model development phase. After a test-retest and inter-observer study, 77 features remained for further analysis, based on a cut-off of 0.85 for the ICC analysis. Further identification of comparable features extracted from the structure merging all metastatic lymph nodes (LN_merged_) to the largest (LN_volume_) or most active node (LN_max_), by means of an intraclass correlation (ICC) over 0.85 and ±10% limits of agreement (LoA) between measurements, was performed (further details in S1 File. Feature pre selection). In summary, 77 features of the primary tumor and 16 from the metastatic lymph nodes were entered in the model development phase.

A prognostic model was fitted to the data with a 10-fold cross-validated least absolute shrinkage and selection operator (LASSO), selecting amongst PET-Radiomics descriptors extracted from the tumor (model 1), LN (model 2) and the union of both structures (model 3) [18]. LASSO selects variables correlated to the measured outcome by shrinking down to zero coefficients weights for features non-related to outcome. Features were entered in the model as continuous variables. Regression coefficients, hazard ratios (HR) and confidence intervals (CI) were estimated using the whole development cohort. A diagram illustrating this methodology is shown in Fig 1. Log-likelihood tests for non-nested models, Akaike information criterion (AIC), were performed to compare the fit of the three derived and independent models. AIC measures the relative quality of model fit to a given cohort, providing substantiation for model selection, i.e. the one with the lower AIC is the preferred model [19]. Log-linearity assumption was verified for the selected features in the final models by fitting a penalized smoothing spline. Cox proportional hazards assumption was graphically examined with the Schoenfeld residuals.

#### Model performance

Model performance was assessed in the development and validation cohorts by means of a concordance-index and corresponding 95% CI [20]. Concordance-index or Harrell’s C-index, evaluates the fraction of patient pairs for which the predicted and actual outcome are concordant, ranging from a random 0.5 to a perfect 1 [21].

All statistical analysis was conducted in R (version 2.15.2), using the libraries: *survival*, *survcomp*, *glmnet*, *cvTols* and *rms* [22]. The development cohort is publicly available at at www.cancerdata.org and Dryad (doi:10.5061/dryad.752153b).

## Results

Table 1 gives a complete overview of patients under analysis including treatment details. Node-positive patients with available FDG-PET-CT scans were included in the analysis: 262 for development and 50 for validation phase. A univariable analysis was performed for each of the clinical variables in the development cohort (Table 2). Results show that TNM staging was not correlated to OS, while N stage was a prognostic factor at 0.1 level (p = 0.09) in the development cohort, with higher stages being associated with a worse prognosis (hazard ratio (HR) stage 2 = 1.44; HR stage 3 = 1.75). The number of LN stations was significantly associated with a higher risk. Radiotherapy dose was also significantly associated with prognosis information. None of the remaining analyzed metrics, including gender, age, histology and chemotherapy showed a significant correlation to OS in our development cohort.

**Table 1:** Demographics and clinical information of development and validation cohorts.

	Development dataset (n = 262)		Validation dataset (n = 50)	
Age				
Mean ± SD	66±10		64±10	
Range	33–86		44–83	
Gender				
Male	172	65.6%	31	62%
Female	90	34.4%	19	38%
Stage				
II	10	3.8%	-	-
IIIa	107	40.8%	32	64%
IIIb	144	55%	18	36%
No information*	1	0.4%	2	4%
N stage				
1	28	10.7%	1	2%
2	151	57.6%	36	72%
3	80	30.5%	6	12%
No information	3	1.2%	7	14%
Number of metastatic LN stations				
Mean ± SD	3.6 ± 2.4		2.1 ± 1.1	
Range	1–12		1–6	
Histology				
Adenocarcinoma	60	22.9%	19	38%
Squamous cell carcinoma	73	27.9%	18	36%
NSCLC-otherwise specified (NOS)	123	46.9%	13	26%
No information	6	2.3%	-	-
Radiotherapy Dose				
Mean ± SD	64.4 ± 7.5		61.8 ± 6.1	
Range	45–99.75**		45–70**	
Chemotherapy				
Yes	227	86.6%	33	66%
No	25	9.6%	-	-
No information	10	3.8%	17	34%

* If no further information about stage was available in the EMD, TNM was reviewed and stage N0 and M1 patients were excluded from analysis

** Only 6 out of the 262 patients from the development dataset and 2 out of the 50 patients in the validation dataset received a dose under 50 Gy. Based on an individual assessment of the medical records of each of these patients, we could find no evidence to justify removing these from the final analysis.

**Table 2:** Univariable Cox regression of clinical variables in development cohort.

Feature	HR	p-value HR	95% CI
Age	0.99	0.10	0.97–1.00
Gender			
Male	Reference		
Female	0.85	0.30	0.63–1.15
Stage			
II	Reference		
IIIa	1.05	0.92	0.48–2.28
IIIb	1.06		0.49–2.28
N stage			
1	Reference		
2	1.44	0.09	0.86–2.40
3	1.75		1.02–2.99
Number of metastatic LN stations			
1	Reference		
2	2.08	<0.01	1.30–3.30
3	1.65		0.98–2.99
≥4	1.95		1.28–2.98
Histology			
Squamous cell carcinoma	Reference		
Adenocarcinoma	0.93	0.18	0.61–1.42
NSCLC-otherwise specified (NOS)	1.26		0.89–1.78
Radiotherapy Dose	0.98	0.03	0.96–0.99
Chemotherapy			
No	Reference		
Yes	1.01	0.11	0.99–1.03

Hazard Ratios (HR) and corresponding p-values and 95% confidence intervals (CI)

**Fig 2:**
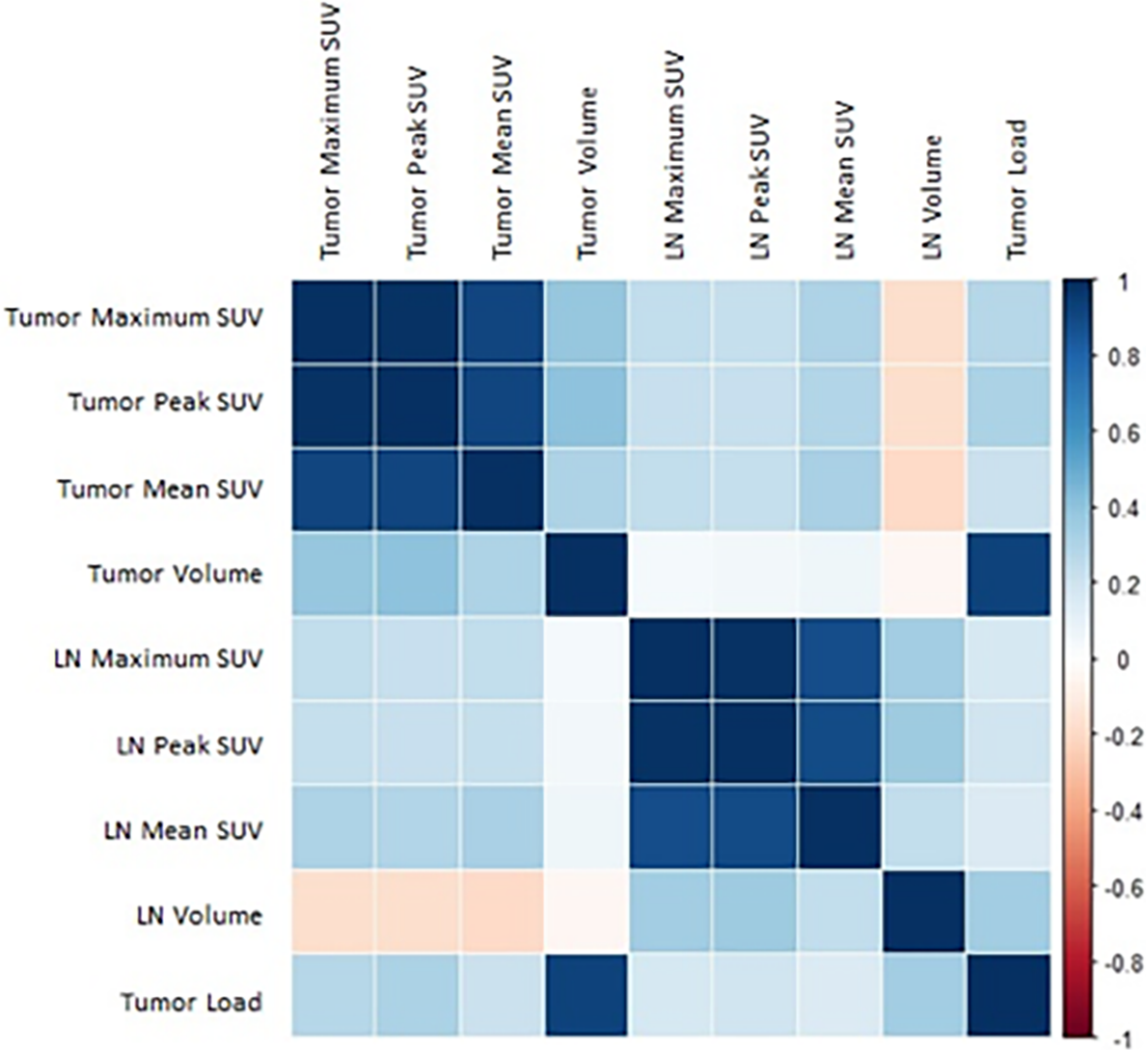
Pearson correlation plot for metabolic descriptors and volume of primary tumor and metastatic lymph nodes in the development dataset.

Results of univariable Cox regression of FDG-PET Radiomics features extracted from both tumor and LNs are shown in the appendix (S2 File. Tables). Only short run emphasis from the texture GLRLM group was significantly correlated to OS when extracted from the primary tumor in the development set. On the other hand, metrics derived from LN showed a good univariable correlation to outcome, with 13 of the 16 analyzed features being significantly related to overall survival. Table 3 displays partial results of this analysis, for which a high Pearson correlation between metabolic features within each structure could be verified, but neither a correlation could be found with own volume, nor with metabolic features of the other structure (Fig 2).

**Table 3:** Distribution of common PET descriptors (maximum, peak and mean) and volume of the primary tumor and LNs.

Structure	Features	Range (Mean ± SD)	HR	p-value HR	95% CI HR	c-index	95% CI c-index
Primary Tumor	Maximum SUV	1.0–32.5 (10.7±5.7)	1.00	0.95	0.97–1.03	0.51	0.40–0.58
	Peak SUV	0.8–29.5 (8.6±4.9)	1.00	0.92	0.97–1.03	0.51	0.40–0.58
	Mean SUV	0.3–15.6 (4.4±2.3)	0.99	0.73	0.92–1.06	0.53	0.44–0.62
	Volume	0.3–702.4 (79.5±104.6)	1.00	0.47	1.00–1.00	0.51	0.43–0.60
Metastatic Lymph Nodes	Maximum SUV	1.2–39.8 (8.3±5.4)	1.05	<0.01	1.02–1.08	0.58	0.49–0.67
	Peak SUV	1.0–32.1 (6.4±4.4)	1.06	<0.01	1.03–1.10	0.58	0.49–0.66
	Mean SUV*	0.5–14.8 (3.5±1.9)	1.14	<0.01	1.06–1.23	0.57	0.48–0.66
	Volume	0.7–325.9 (35.3±42.9)	1.01	<0.01	1.00–1.01	0.60	0.51–0.68
	Tumor Load	3.8–709.6 (114.8±111.3)	1.01	0.03	1.00–1.01	0.58	0.49–0.66

Univariable Cox regression of common FDG-PET descriptors extracted from primary tumor and metastatic lymph nodes of the development cohort: Hazard Ratios (HR) and corresponding p-values and 95% confidence intervals (CI); univariable performance expressed by concordance-index (c-index) and associated 95% CI.

* Mean SUV is a generalization of the mean SUV distribution across all independent metastatic lymph nodes, as extracted from a structure merging all nodes. Total load refers to the combined volume of the primary tumor and metastatic lymph nodes.

**Fig 3:**
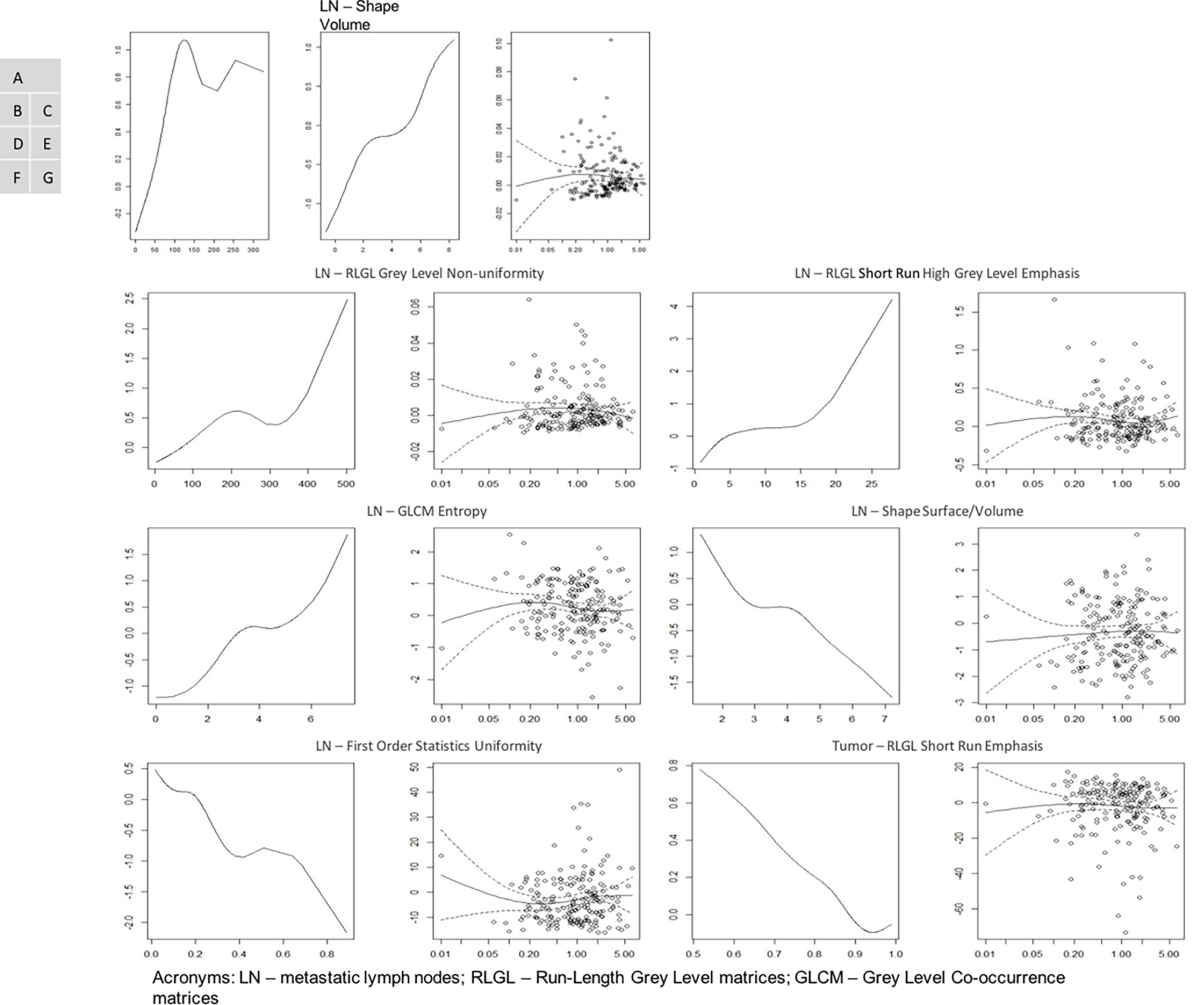
Log-linear and proportional hazards assumptions verification. Graphically, log-linearity was verified by fitting a penalised smoothing spline on the univariable effect of each variable included in models (left graph), while proportional hazards were analysed by plotting Schoenfeld residuals versus log (time) (right graph). These included variables for LN, the (A) volume, (B) GLRLM grey level non-uniformity, (C) GLRLM short run high grey level emphasis, (D) GLCM entropy, (E) surface to volume ratio, and (F) uniformity, and (G) GLRLM short run emphasis of tumour. All variables were log (linear), except LN volume (A left), for which a logarithmic transformation was performed (A middle). All variables satisfied the proportional hazards assumption. Automatic feature selection for model 1 (based solely on primary tumor imaging features) converged to a single metric of the GLRLM group—short run emphasis, with a C-index of 0.53 (95% confidence interval [CI] = 0.49–0.58) and an external validation of 0.54. Model 2 (based on imaging features from LN) included total volume and the surface to volume ratio (shape), histogram uniformity (first order statistics), grey level non-uniformity and short run high grey level emphasis (GLRLM of the textural group), reaching a C-index of 0.62 (95% CI = 0.57–0.66) with an external validation of 0.56. Important to note that LN volume is an independent prognostic metric, with an univariable performance of 0.60 (95% CI = 0.51–0.68). Finally, model 3 selected the same feature as model 1 and four features from the LN, replacing short run high grey level emphasis–GLRLM, by entropy–GLCM, and reached a performance of 0.62 (95% CI = 0.58–0.67), and 0.59 in the external cohort. No metrics from the IVH sub-category were selected from any of the analyzed structures for the derived models. Based on an AIC test, model 3 (1854.5) was shown to be a better fit than model 2 (1857.4), which itself was already a more precise fit compared to model 1 (1876.4). In summary, the addition of nodal imaging information resulted in a better model fit, compared to a model based exclusively on features derived from the primary tumor.

Three model approaches were derived and fitted to the data in the development cohort. These are represented in Table 4, with corresponding hazard ratios and concordance-index. Selected features were log-linear, except for LN volume, which had to be converted into a logarithmic scale. The proportional hazards assumption was satisfied for all features. Graphical assessment of these assumptions can be appreciated from Fig 3.

**Table 4:** Distribution of features included in the Cox regression model for FDG-PET-CT-based features extracted from pre-radiotherapy scans of NSCLC patients.

			Tumor and nodes separately			Tumor and nodes combined		
Model	Features	Range (Mean ± SD)	Hazard Ratios	p-value	C-index [95% CI]	Hazard Ratios	p-value	C-index [95% CI]
Primary Tumor	GLRLM–Short Run Emphasis	0.52–0.99 (0.89±0.07)	0.13	0.04	0.53 [0.49–0.58] 0.54**	0.06	0.01	0.62 [0.58–0.67] 0.59**
Metastatic Lymph Nodes	Shape–Volume*	0.65–325.9 (35.3±42.9)	0.93	0.47	0.62 [0.57–0.66] 0.56**	0.88	0.28	
	GLRLM–Grey Level Non-uniformity	3.12–501.6 (68.5±75.4)	1.00	0.02		1.00	0.02	
	GLRLM–Short Run High Grey Level Emphasis	0.86–27.8 (5.76±3.55)	1.03	0.83		-	-	
	GLCM–Entropy	0.00–7.37 (3.82±1.23)	-	-		1.17	0.48	
	Shape–Surface/Volume	1.33–27.8 (5.76±3.55)	0.90	0.41		0.94	0.67	
	Stats–Uniformity	0.02–0.89 (0.17±0.12)	0.10	0.06		0.08	0.19	

Analysis was conducted for primary tumor and metastatic lymph nodes separately, and for both structures in combination. Hazard Ratios (HR) and corresponding p-values are reported. Performance of the model is expressed by internal and external** concordance-index (C-index). Internal performance includes associated 95% confidence-interval (CI) of the C-index.

Acronyms: GLCM–Grey Level Co-occurrence matrices; GLRLM–Grey Level Run-length matrices; Stats–first order statistics

* A logarithmic transformation was applied to LN volume

** External validation

## Discussion

Disease management of NSCLC is a primary concern, for which prognostic assessment is essential to fulfil the potential of individualized and personalized treatment. Nowadays, a wide range of information sources are available of which the non-invasive type play a fundamental role in reducing the patients’ burden [4–6]. Among these, metabolic imaging has been increasingly explored for prognosis assessment, based on SUV patterns of FDG of the primary tumor, as an extension of its primary diagnostic function: detection of metastatic lymph nodes and distant metastasis [23, 24].

NSCLC patients often present with lymph node involvement at diagnosis, which deeply impacts prognosis and response to treatment [1, 2]. Apart from the number of metastatic lymph node stations, lymph node size and corresponding metabolic activity may vary among patients [25]. Diagnosis of metastatic hilar/mediastinal lymph nodes is commonly performed through FDG-PET-CT and consecutive EBUS/EUS or mediastinoscopy [26–30]. Diffusion-weighted magnetic resonance imaging (DWI) is also a solid alternative for lymph node detection, as proven by the work of Shen et. al [31]. In the work carried out, given the availability of FDG-PET-CT scans for all patients under analysis as part of their protocol for radiotherapy treatment, we proceeded the analysis with lymph nodes detected using this imaging modality. Based on the rationale that disease progression and the ability to metastasize are closely related to the presence of metastatic lymph nodes, we hypothesized that FDG-PET-based Radiomics information of these nodes would provide additional prognostic information to the information that is obtained from the primary tumor [23]. Radiomics has been proven to have prognostic potential in predicting clinical outcomes or treatment monitoring in different cancer types [13, 16, 32, 33] and can essentially be applied to different medical imaging modalities and disease-related structures such as the primary tumor, metastatic lymph nodes or metastatic lesions.

Common clinical metrics associated with disease prognosis, including TNM staging (p = 0.92), could not be attributed statistical significance in the development cohort. Nevertheless, patients were staged in accordance to later editions of the classification system, that newer classification as updated by the 8^th^ edition may overcome [34]. On the other hand, higher number of metastatic lymph nodes could be associated with a statistical significant higher risk (p<0.01), already anticipating the extra information these structures may provide, that we further evaluated through imaging descriptors.

Given the broad range of imaging descriptors analyzed by a Radiomics approach, an initial step in this analysis was to perform an exploratory univariable analysis of the most commonly analyzed PET metrics when extracted from tumor and LNs, namely maximum, peak, and mean SUV, volume and tumor load. None of the metabolic metrics extracted from tumor had significant prognostic value, whereas the same ones extracted from LNs were related to OS and yielded an univariable C-index of at least 0.57 (Table 3). No statistical significance could be associated with tumor volume (p = 0.47), as opposed to LN volume (p<0.01; c-index 0.60, 95% CI = 0.51–0.68). Tumor load, sum of tumor and LNs volumes, also had prognostic value, in line with previous studies [35]. Despite the strong correlation between metabolic features within each structure, neither a correlation with own volume, nor with metabolic features of the other structure could be found, contrary to previous evidence of correlation between maximum SUV of tumor and total LN volume [36]. One can already infer from this univariable analysis the considerable prognostic value that the metabolic distribution within metastatic lymph nodes has for node-positive NSCLC patients, which is further reinforced by remaining features.

The main purpose of this study was to analyze imaging features extracted from the LNs and compare it against the ones extracted from the primary tumor. In addition the non-univariable statistical significance attributed to common clinical features (e.g. TNM staging), subsequent modeling conducted solely on FDG-PET imaging features. Prior to development of the three prognostic models, a pre-selection of the imaging features, comprising their stability and robustness as discussed by Leijenaar et al. [14], and supported by Desseroit et al. [37], was conducted and is discussed in the appendix section. Of the three derived independent fits, the model with features from LNs alone or LNs in combination with primary tumor performed best (c-index 0.62). The model included at least one feature from categories of shape and size, first order statistics and texture descriptor, excluding features from the IVH group, despite their proven univariable prognostic evidence in a previous study [38]. The intrinsic heterogeneity described by the FDG distribution within a primary tumor has proven its prognostic power, not only for NSCLC, but also for other cancer types [39, 40]. Inclusion of such descriptors in derived prognostic models, gathering heterogeneity insight into not only primary tumor but also metastatic lymph nodes, resulted in a model with a better fit and more accurate description of disease structures. Shape descriptors, including total LN volume, included in the multivariable model, were shown to be an independent prognostic parameter. In a previous study, tumor load was revealed to be a prognostic factor, but not LN volume independently, which we proved in this study [35]. Finally, and despite their univariate prognostic value, particularly when assessed from LN, none of the most common SUV descriptors were included in the final models. However, as Radiomics analysis includes a large number of features, selection of the most promising ones is difficult. We attempted to overcome this difficulty using an automatic feature selection routine (LASSO), to ensure an optimal exclusion of redundant or highly correlated features from the final models derived, further complemented with and external validation. Nonetheless, it cannot be completely excluded that other variables have similar or even greater prognostic value than the current ones, and therefore larger imaging datasets are needed to validate and confirm our findings. Similarly, another limitation of our study was the lack of additional patient data for the validation phase. A lower performance is commonly observed when validating a model against new, independent, and external cohorts, which is most frequently attributed to discrepancies between development and validation data [41]. A larger validation cohort would increase the robustness of the validation procedure. Nevertheless, we observed a benefit from combining imaging features from both primary tumor and metastatic lymph nodes for node-positive NSCLC patients. In terms of prognostics, this should not be disregarded, particularly when compared to the limited capacity of humans to infer an accurate prognosis from same data [42]. In fact, the most commonly accepted standard for disease prognosis assessment failed to provide a significant stratification of risk patients (TNM stage).

A recent study with 139 NSCLC patients treated with at least 60Gy with a concurrent (chemo)radiotherapy regimen showed the importance of measuring the post-treatment SUV in the metastatic lymph nodes, as an increase in both the absolute value and percentage of residual activity compared to the pre-treatment SUV were associated with worse local-regional control [43]. In our study we looked only at the pre-treatment scan and could already discern an association with OS of imaging features derived from the metastatic lymph nodes that could not be assessed based on the primary tumor for node-positive patients. However, it is hypothesized that the the variation of FDG-PET-Radiomics features between subsequent scans at an early phase of treatment for both the primary tumor and the metastatic lymph nodes, and their impact on survival for NSCLC patients as a complement to the positive findings reported here. Also, a positive correlation between PET information derived from LNs and overall relapse has been reported [44]. Our analysis focused primarily on overall survival, and therefore we could not validate these findings. In the future we will be able to analyze this outcome as we are currently improving our data collection routines, to further evaluate other outcomes, actually limited to overall survival. Nevertheless, our findings emphasize the importance of analyzing FDG-PET signal of metastatic lymph nodes prior to radiotherapy, to further complement the information retrieved from the primary tumor.

Based on the positive and relevant findings we documented, we have plans to extend our analysis in a similar manner to other disease sites, particularly head and neck cancer, for which the involvement of the nodes is a well-known prognostic risk factor [45]. Likewise, we are also aiming to analyze other PET tracers, particularly hypoxia markers (e.g. HX4) and compare the results with FDG uptake [46, 47]. Further, it has been recently shown that combining Radiomics from both FDG-PET and low-dose CT can improve prognosis in NSCLC [48], therefore, combining Radiomics from LNs extracted from the CT part of the FDG-PET-CT would be a much desirable and natural step for future analysis with larger datasets. Seemingly, other endpoints could obviously be analyzed following this Radiomics approach, namely FDG-PET-Radiomics signal correlation to tumor marker. This rationale follows the correlation demonstrated between maximum SUV and an increased expression of glucose transporters 1 and p53 for adenocarcinoma NSCLC patients, but not squamous cell by the work of Taylor et al. [49]. As pointed out, these studies need to take into consideration the effect of different tumor histology types, which correction was not performed in this work. Resistance of p53-related chemotherapy has also been shown to be linked with maximum SUV by the work of Duan et al. [50]. Correlation to therapy response was however not the subject of this study, as the endpoint under analysis was overall survival due to any cause, but one can definitely leave for future approaches the link between Radiomics descriptors and tumor markers related to response to therapy.

In summary, common SUV descriptors derived from metastatic lymph nodes were associated with overall survival in a large cohort of NSCLC patients. Additionally, PET information demonstrated to have higher prognostic value when extracted from metastatic lymph nodes in comparison to the primary tumor alone, further complementing its information. The use of 3D information based on imaging is becoming a broader field with expected great gain for patients’ outcomes assessment and treatment planning adaptations, following its application to dissimilar structures as primary tumor, metastatic lymph nodes and possibly distant metastasis.

## Supporting information

S1 File Feature pre selection. Methodology conducted for feature pre-selection for both primary tumor (tumor) and metastatic lymph nodes (LN), and corresponding results and interpretation.
(DOCX)

S2 File Tables. Univariable analysis of stable and robust Radiomics features from primary tumor for development and validation datasets and that were common surrogates as extracted from the merged and largest node as well as the merged and most active node, and therefore entered as continuous variables in the multivariable model building.
(DOCX)
