# Supplementary material for: 18F-fluorodeoxyglucose positron-emission tomography (FDG-PET)-Radiomics of metastatic lymph nodes and primary tumor in non-small cell lung cancer (NSCLC) – A prospective externally validated study
Source: PLoS One. 2018 Mar 1;13(3):e0192859. doi: 10.1371/journal.pone.0192859 (PMC5832210; doi:10.1371/journal.pone.0192859)
Supplement: S1 File — Methodology conducted for feature pre-selection for both primary tumor (tumor) and metastatic lymph nodes (LN), and corresponding results and interpretation. (DOCX) [file pone.0192859.s001.docx]

Supporting information to the manuscript “^18^FDG-PET Radiomics of metastatic lymph nodes and primary tumor in NSCLC - 'a prospective externally validated study”

This appendix presents a methodology conducted for feature pre-selection for both primary tumor (tumor) and metastatic lymph nodes (LN), and corresponding results and interpretation.

Robust and stable ^18^F-fluorodeoxyglucose (FDG) Positron Emission Tomography (PET) Radiomics features of the primary tumor were selected from a test-retest and inter-observer analysis, using an intraclass correlation coefficient (ICC) analysis [[1](#_ENREF_1)]. Details of the methodology and corresponding results are presented elsewhere [[2](#_ENREF_2)]. Features presenting an intraclass correlation coefficient (ICC) over 0.85 in both test-retest and multiple observer settings were defined as robust. The selection procedure resulted in a total of 77 features.

Metastatic lymph nodes were analyzed as a single structure combining all the individual metastatic LN stations. This intermediate step was followed for both data reduction (as patients can have a large number of metastatic lymph nodes) and computation simplicity. We further assessed how feature values extracted from this merged structure (LN_merged_) compared to values from the largest (LN_volume_) or most active LN (LN_max_), regardless of the corresponding lymph node station. To this end, we randomly selected a subgroup of 88 patients (34%) of the 262 patients in the derivation dataset with lymphadenopathy and identified LN_volume_ and LN_max_, from which the robust and stable PET Radiomics were extracted. These same features were then derived from the radiotherapy planning structure comprising the total nodal volume (LN_merged_). The intraclass correlation coefficient (ICC) was calculated between features extracted from LN_volume_ and LN_merged_ (A), as well as between features extracted from LN_max_ and LN_merged_ (B). The ICC is based on the analysis of variance and assumes values between 0 and 1, and were further adjusted as by Spearman-Brown, to reflect the means of the different ratters – ICC(1,k)[[1](#_ENREF_1)]. The ICC can be large only if there is no bias and the paired measurements are in close agreement. Hence, we extended comparisons A and B with a 95% limit of agreement (LoA), based on the methods of Bland and Altman [[3](#_ENREF_3), [4](#_ENREF_4)].

The largest and most active LN was the same for 55 out of the 88 (62.5%) patients in the sub-analysis. For LN_volume_ and LN_merged_, 37 out of the 77 features yielded an ICC(1,k) over 0.85 and a LoA within the ±10% interval, while for LN_max_ and LN_merged_ this number decreased to 26 features (Table 1), that were condensed in combination into 12 common features between structures. This analysis followed the aim to capture the independent contributions of each independent node in a single feature value, which varies among patients. Some of the derived features are dependent on the Region-of-interest (ROI), that when comprising more than a single independent lymph node, may influence its value. For this reason, we aimed to identify features that, extracted from a single structure, would be comparable if extracted from the largest or most active LN, improving its interpretability. In addition to these features, we included maximum and peak SUV as well as total LN volume and tumor load in the multivariable modelling, following the univariable evidence that a single high uptake region and LN volume are prognostic factors (Table 3 in main text), even though these metrics were not gathered from the largest node, but were, obviously, from the most active one.

In summary, a total of 77 stable and robust features from the primary tumor and 16 for LN_merged_, were selected for further analysis following the rationale described.

Table 1 – Number of PET features with an ICC(1,k) over 0.85 and within a ±10% LoA interval as derived from the largest (LN_volume_) or more active node (LN_max_) and merged structure (LN_merged_), for the different groups of features. Number of stable and robust features previously identified are displayed between brackets in the header of each sub-category.

| Structure | Statistics (13/16) | Shape (9/13) | GLRLM (8/11) | GLCM (10/22) | GLSZM (1/11) | IVH  (36/45) | Total (77/118) |
| --- | --- | --- | --- | --- | --- | --- | --- |
| LN_volume_ | 5 | 1 | 6 | 4 | 0 | 21 | 37 |
| LN_max_ | 8 | 1 | 6 | 7 | 0 | 4 | 26 |

Acronyms: GLRLM –grey level run-length; GLCM – grey level co-occurrence; GLSZM – grey level size zone matrices; IVH – intensity-volume histograms.

References

1. Shrout PE, Fleiss JL. Intraclass correlations: uses in assessing rater reliability. Psychological bulletin. 1979;86(2):420-8. Epub 1979/03/01. PubMed PMID: 18839484.

2. Leijenaar RT, Carvalho S, Velazquez ER, van Elmpt WJ, Parmar C, Hoekstra OS, et al. Stability of FDG-PET Radiomics features: an integrated analysis of test-retest and inter-observer variability. Acta Oncol. 2013;52(7):1391-7. Epub 2013/09/21. doi: 10.3109/0284186x.2013.812798. PubMed PMID: 24047337; PubMed Central PMCID: PMCPmc4533992.

3. Bland JM, Altman DG. Statistical methods for assessing agreement between two methods of clinical measurement. Lancet (London, England). 1986;1(8476):307-10. Epub 1986/02/08. PubMed PMID: 2868172.

4. Dewitte K Fau - Fierens C, Fierens C Fau - Stockl D, Stockl D Fau - Thienpont LM, Thienpont LM. Application of the Bland-Altman plot for interpretation of method-comparison studies: a critical investigation of its practice. (0009-9147 (Print)).
