# Supplementary material for: 18F-fluorodeoxyglucose positron-emission tomography (FDG-PET)-Radiomics of metastatic lymph nodes and primary tumor in non-small cell lung cancer (NSCLC) – A prospective externally validated study
Source: PLoS One. 2018 Mar 1;13(3):e0192859. doi: 10.1371/journal.pone.0192859 (PMC5832210; doi:10.1371/journal.pone.0192859)
Supplement: S2 File — Univariable analysis of stable and robust Radiomics features from primary tumor for development and validation datasets and that were common surrogates as extracted from the merged and largest node as well as the merged and most active node, and therefore entered as continuous variables in the multivariable model building. (DOCX) [file pone.0192859.s002.docx]

Supporting information to the manuscript “^18^FDG-PET Radiomics of metastatic lymph nodes and primary tumor in NSCLC - 'a prospective externally validated study”

The results presented in this appendix, while not essential to the main message of the manuscript, further complement it. Tables 1 and 3 present the univariable analysis of stable and robust Radiomics features from primary tumor for development and validation datasets. Tables 2 and 4 present the univariable analysis of stable and robust PET Radiomics features that were common surrogates as extracted from the merged and largest node as well as the merged and most active node, and therefore entered as continuous variables in the multivariable model building.

Table 1 – Univariable Cox regression on the robust and stable features of the primary tumor from the development dataset (n=262).

| Class of feature | Feature | Hazard Ratio | p-value | 95% Confidence Interval | C-index |
| --- | --- | --- | --- | --- | --- |
| Shape and size  (13/16) | Compactness | 1.050 | 0.457 | 0.924 - 1.193 | 0.511 |
|  | Maximum Diameter 2Dx | 1.017 | 0.468 | 0.971 - 1.066 | 0.508 |
|  | Maximum Diameter 2Dy | 1.013 | 0.580 | 0.967 - 1.062 | 0.510 |
|  | Maximum Diameter 2Dz | 1.033 | 0.232 | 0.980 - 1.088 | 0.524 |
|  | Maximum Diameter 3D | 1.032 | 0.140 | 0.990 - 1.076 | 0.529 |
|  | Surface/Volume | 0.988 | 0.850 | 0.873 - 1.119 | 0.508 |
|  | Surface | 1.001 | 0.302 | 0.999 - 1.002 | 0.515 |
|  | Volume | 1.000 | 0.470 | 0.999 - 1.002 | 0.514 |
|  | Number of voxels | 1.000 | 0.397 | 0.999 - 1.000 | 0.519 |
| First Order Statistics  (9/13) | Energy | 1.000 | 0.379 | 0.999 - 1.000 | 0.500 |
|  | Entropy | 0.922 | 0.305 | 0.790 - 1.077 | 0.516 |
|  | Maximum | 1.001 | 0.950 | 0.975 - 1.028 | 0.491 |
|  | Mean Deviation | 0.979 | 0.761 | 0.854 - 1.123 | 0.518 |
|  | Mean | 0.988 | 0.732 | 0.924 - 1.057 | 0.527 |
|  | Median | 0.989 | 0.736 | 0.924 - 1.057 | 0.528 |
|  | Range | 1.001 | 0.914 | 0.975 - 1.028 | 0.494 |
|  | Root Mean Square | 0.990 | 0.736 | 0.933 - 1.050 | 0.524 |
|  | Standard Deviation | 0.984 | 0.788 | 0.875 - 1.107 | 0.517 |
|  | Peak SUV | 1.002 | 0.916 | 0.972 - 1.032 | 0.490 |
|  | Total Energy | 1.000 | 0.452 | 0.999 - 1.000 | 0.498 |
|  | Uniformity | 2.883 | 0.095 | 0.831 - 9.998 | 0.518 |
|  | Variance | 1.003 | 0.670 | 0.988 - 1.019 | 0.483 |
| Texture  (19/44) | Grey Level Run-length (GLRLM)  (8/11) | | | | |
|  | Grey Level Non-uniformity | 1.000 | 0.347 | 0.999 - 1.001 | 0.525 |
|  | High Grey Level Run Emphasis | 1.000 | 0.662 | 0.999 - 1.001 | 0.480 |
|  | Long Run Emphasis | 1.110 | 0.076 | 0.989 - 1.246 | 0.529 |
|  | Long Run High Grey Level Emphasis | 1.000 | 0.713 | 0.999 - 1.001 | 0.478 |
|  | Run-length Non-uniformity | 1.000 | 0.487 | 0.999 - 1.000 | 0.514 |
|  | Run Percentage | 0.207 | 0.055 | 0.042 - 1.033 | 0.531 |
|  | Short Run Emphasis | 0.131 | 0.043 | 0.018 - 0.941 | 0.533 |
|  | Short Run High Grey Level Emphasis | 0.995 | 0.790 | 0.961 - 1.031 | 0.520 |
|  | Grey Level Co-occurrence (GLCM)  (10/22) | | | | |
|  | Autocorrelation | 1.000 | 0.682 | 0.999 - 1.001 | 0.479 |
|  | Cluster Prominence | 1.000 | 0.463 | 0.999 - 1.000 | 0.489 |
|  | Cluster Tendency | 1.000 | 0.655 | 0.999 - 1.001 | 0.488 |
|  | Contrast | 1.001 | 0.894 | 0.991 - 1.010 | 0.477 |
|  | Dissimilarity | 0.964 | 0.512 | 0.864 - 1.076 | 0.526 |
|  | Entropy | 0.979 | 0.697 | 0.878 - 1.091 | 0.512 |
|  | Sum Average | 0.998 | 0.768 | 0.982 - 1.013 | 0.523 |
|  | Sum Entropy | 0.973 | 0.753 | 0.821 - 1.153 | 0.511 |
|  | Sum of Squares | 1.000 | 0.691 | 0.999 - 1.001 | 0.478 |
|  | Sum of Variance | 1.000 | 0.643 | 0.999 - 1.000 | 0.478 |
|  | Grey Level Size Zone (GLSZM)  (1/11) | | | | |
|  | High Intensity Large Area Emphasis | 1.000 | 0.958 | 0.999 - 1.000 | 0.502 |
| Intensity-Volume Histograms (IVH)  (36/45) | Absolute Intensity of Relative Volume (AIRV)  (9/9) | | | | |
|  | AIRV 10% | 0.996 | 0.824 | 0.958 - 1.034 | 0.519 |
|  | AIRV 20% | 0.992 | 0.724 | 0.949 - 1.037 | 0.525 |
|  | AIRV 30% | 0.990 | 0.681 | 0.941 - 1.040 | 0.528 |
|  | AIRV 40% | 0.987 | 0.669 | 0.932 - 1.046 | 0.530 |
|  | AIRV 50% | 0.989 | 0.736 | 0.924 - 1.057 | 0.528 |
|  | AIRV 60% | 0.987 | 0.741 | 0.911 - 1.068 | 0.529 |
|  | AIRV 70% | 0.982 | 0.705 | 0.891 - 1.081 | 0.527 |
|  | AIRV 80% | 0.982 | 0.763 | 0.870 - 1.107 | 0.522 |
|  | AIRV 90% | 0.981 | 0.809 | 0.836 - 1.150 | 0.519 |
|  | Absolute Volume of Relative Intensity (AVRI)  (9/9) | | | | |
|  | AVRI 10% | 1.000 | 0.577 | 0.999 - 1.002 | 0.513 |
|  | AVRI 20% | 1.000 | 0.713 | 0.999 - 1.002 | 0.512 |
|  | AVRI 30% | 1.000 | 0.873 | 0.998 - 1.002 | 0.509 |
|  | AVRI 40% | 1.000 | 0.994 | 0.998 - 1.002 | 0.497 |
|  | AVRI 50% | 1.000 | 0.967 | 0.996 - 1.004 | 0.497 |
|  | AVRI 60% | 1.001 | 0.854 | 0.994 - 1.007 | 0.494 |
|  | AVRI 70% | 1.001 | 0.872 | 0.989 - 1.013 | 0.485 |
|  | AVRI 80% | 0.999 | 0.956 | 0.960 - 1.039 | 0.522 |
|  | AVRI 90% | 0.958 | 0.701 | 0.770 - 1.192 | 0.512 |
|  | Mean Intensity of Relative Volume (MIRV)  (9/9) | | | | |
|  | MIRV 10% | 0.996 | 0.818 | 0.963 - 1.031 | 0.519 |
|  | MIRV 20% | 0.995 | 0.792 | 0.958 - 1.033 | 0.520 |
|  | MIRV 30% | 0.994 | 0.761 | 0.954 - 1.035 | 0.521 |
|  | MIRV 40% | 0.993 | 0.743 | 0.951 - 1.037 | 0.523 |
|  | MIRV 50% | 0.992 | 0.743 | 0.947 - 1.039 | 0.524 |
|  | MIRV 60% | 0.992 | 0.738 | 0.944 - 1.042 | 0.525 |
|  | MIRV 70% | 0.991 | 0.737 | 0.940 - 1.045 | 0.525 |
|  | MIRV 80% | 0.990 | 0.732 | 0.935 - 1.048 | 0.526 |
|  | MIRV 90% | 0.989 | 0.735 | 0.930 - 1.052 | 0.526 |
|  | Total Lesion Glycolysis of Relative Intensity (TLGRI)  (9/9) | | | | |
|  | TLGRI 10% | 1.000 | 0.600 | 0.999 - 1.000 | 0.505 |
|  | TLGRI 20% | 1.000 | 0.649 | 0.999 - 1.000 | 0.504 |
|  | TLGRI 30% | 1.000 | 0.713 | 0.999 - 1.000 | 0.502 |
|  | TLGRI 40% | 1.000 | 0.744 | 0.999 - 1.000 | 0.498 |
|  | TLGRI 50% | 1.000 | 0.623 | 0.999 - 1.000 | 0.494 |
|  | TLGRI 60% | 1.000 | 0.466 | 1.000 - 1.001 | 0.490 |
|  | TLGRI 70% | 1.000 | 0.406 | 1.000 - 1.001 | 0.484 |
|  | TLGRI 80% | 1.001 | 0.396 | 0.999 - 1.003 | 0.479 |
|  | TLGRI 90% | 1.004 | 0.556 | 0.992 - 1.015 | 0.485 |

Table 2 - Univariable Cox regression on the robust and stable features of the metastatic lymph nodes from the development dataset (n=262). Analysis was conducted for features with an ICC(1,k) over 0.85 and within a ±10% LoA interval as derived from the largest (LN_volume_) and more active node (LN_max_) and merged structure (LN_merged_), for the different groups of features, with the exception of the ones indicated by an asterisk (*). Volume corresponds to the total volume of all metastatic lymph nodes (^†^).

| Class of feature | Feature | Hazard Ratio | p-value | 95% Confidence Interval | C-index |
| --- | --- | --- | --- | --- | --- |
| Shape and size (1/13) | Volume^†^ | 1.006 | <0.01 | 1.004 - 1.009 | 0.599 |
|  | Surface/Volume | 0.717 | <0.01 | 0.611 - 0.841 | 0.585 |
| First Order Statistics  (2/16) | Entropy | 1.358 | <0.01 | 1.162 - 1.586 | 0.585 |
|  | Maximum* | 1.051 | <0.01 | 1.024 - 1.078 | 0.580 |
|  | Mean* | 1.143 | <0.01 | 1.060 - 1.233 | 0.572 |
|  | Peak SUV* | 1.064 | <0.01 | 1.031 - 1.078 | 0.580 |
|  | Uniformity | 0.057 | <0.01 | 0.013 - 0.246 | 0.587 |
| Texture  (6/44) | Grey Level Run-length (GLRLM) | | | | |
|  | Grey Level Non-uniformity | 1.003 | <0.01 | 1.001 - 1.005 | 0.569 |
|  | Long Run Emphasis | 0.942 | 0.601 | 0.752 - 1.179 | 0.539 |
|  | Short Run Emphasis | 9.561 | 0.061 | 0.904 - 101.1 | 0.546 |
|  | Short Run High Grey Level Emphasis | 1.080 | <0.01 | 1.039 - 1.124 | 0.582 |
|  | Grey Level Co-occurrence (GLCM) | | | | |
|  | Entropy | 1.290 | <0.01 | 1.143 - 1.455 | 0.593 |
|  | Sum of Average | 1.034 | <0.01 | 1.017 - 1.052 | 0.580 |
| IVH  (3/45) | Absolute Volume of Relative Intensity (AVRI) | | | | |
|  | AVRI 90% | 1.428 | 0.070 | 0.971 - 2.100 | 0.545 |
|  | Mean Intensity of Relative Volume (MIRV) | | | | |
|  | MIRV 10% | 1.067 | <0.01 | 1.030 - 1.106 | 0.575 |
|  | MIRV 20% | 1.076 | <0.01 | 1.034 - 1.121 | 0.575 |

Table 3 – Univariable Cox regression on the robust and stable features of the primary tumor from the validation dataset (n=50).

| Class of feature | Feature | Hazard Ratio | p-value | 95% Confidence Interval | C-index |
| --- | --- | --- | --- | --- | --- |
| Shape and size  (13/16) | Compactness | 1.199 | 0.293 | 0.850 - 1.680 | 0.553 |
|  | Maximum Diameter 2Dx | 1.037 | 0.576 | 0.910 - 1.180 | 0.533 |
|  | Maximum Diameter 2Dy | 1.107 | 0.088 | 0.980 - 1.240 | 0.576 |
|  | Maximum Diameter 2Dz | 1.097 | 0.216 | 0.950 - 1.270 | 0.565 |
|  | Maximum Diameter 3D | 1.079 | 0.202 | 0.960 - 1.210 | 0.561 |
|  | Surface/Volume | 0.939 | 0.810 | 0.560 - 1.570 | 0.545 |
|  | Surface | 1.003 | 0.098 | 1.000 - 1.010 | 0.549 |
|  | Volume | 1.003 | 0.092 | 1.000 - 1.010 | 0.551 |
|  | Number of voxels | 1.000 | 0.175 | 0.999 - 1.000 | 0.549 |
| First Order Statistics  (9/13) | Energy | 1.000 | 0.242 | 0.999 - 1.000 | 0.555 |
|  | Entropy | 1.082 | 0.674 | 0.750 - 1.560 | 0.561 |
|  | Maximum | 1.023 | 0.217 | 0.990 - 1.060 | 0.598 |
|  | Mean Deviation | 1.044 | 0.635 | 0.870 - 1.250 | 0.583 |
|  | Mean | 1.000 | 0.992 | 0.900 - 1.100 | 0.500 |
|  | Median | 0.978 | 0.696 | 0.880 - 1.090 | 0.535 |
|  | Range | 1.025 | 0.279 | 0.980 - 1.070 | 0.594 |
|  | Root Mean Square | 1.023 | 0.213 | 0.990 - 1.060 | 0.605 |
|  | Standard Deviation | 1.007 | 0.881 | 0.920 - 1.100 | 0.519 |
|  | Peak SUV | 1.053 | 0.514 | 0.900 - 1.230 | 0.590 |
|  | Total Energy | 1.000 | 0.280 | 0.999 - 1.000 | 0.552 |
|  | Uniformity | 0.389 | 0.680 | 0.000 - 34.55 | 0.544 |
|  | Variance | 1.004 | 0.648 | 0.990 - 1.020 | 0.590 |
| Texture  (19/44) | Grey Level Run-length (GLRLM)  (8/11) | | | | |
|  | Grey Level Non-uniformity | 1.002 | 0.283 | 1.000 - 1.010 | 0.533 |
|  | High Grey Level Run Emphasis | 1.000 | 0.901 | 0.999 - 1.000 | 0.550 |
|  | Long Run Emphasis | 0.950 | 0.771 | 0.670 - 1.340 | 0.460 |
|  | Long Run High Grey Level Emphasis | 1.000 | 0.967 | 0.999 - 1.000 | 0.547 |
|  | Run-length Non-uniformity | 1.000 | 0.220 | 0.999 - 1.000 | 0.552 |
|  | Run Percentage | 0.960 | 0.980 | 0.040 - 22.47 | 0.543 |
|  | Short Run Emphasis | 1.241 | 0.915 | 0.020 - 64.63 | 0.460 |
|  | Short Run High Grey Level Emphasis | 1.001 | 0.973 | 0.950 - 1.060 | 0.526 |
|  | Grey Level Co-occurrence (GLCM)  (10/22) | | | | |
|  | Autocorrelation | 1.000 | 0.988 | 0.999 - 1.000 | 0.539 |
|  | Cluster Prominence | 1.000 | 0.428 | 0.999 - 1.000 | 0.616 |
|  | Cluster Tendency | 1.000 | 0.530 | 0.999 - 1.000 | 0.598 |
|  | Contrast | 1.001 | 0.739 | 0.999 - 1.000 | 0.537 |
|  | Dissimilarity | 1.013 | 0.819 | 0.910 - 1.130 | 0.517 |
|  | Entropy | 1.035 | 0.818 | 0.770 - 1.390 | 0.539 |
|  | Sum Average | 0.999 | 0.939 | 0.980 - 1.020 | 0.479 |
|  | Sum Entropy | 1.157 | 0.566 | 0.700 - 1.910 | 0.567 |
|  | Sum of Squares | 1.000 | 0.960 | 0.999 - 1.000 | 0.538 |
|  | Sum of Variance | 1.000 | 0.971 | 0.999 - 1.000 | 0.541 |
|  | Grey Level Size Zone (GLSZM)  (1/11) | | | | |
|  | High Intensity Large Area Emphasis | 1.000 | 0.933 | 0.999 - 1.000 | 0.493 |
| Intensity-Volume Histograms (IVH)  (36/45) | Absolute Intensity of Relative Volume (AIRV)  (9/9) | | | | |
|  | AIRV 10% | 1.009 | 0.740 | 0.960 - 1.060 | 0.549 |
|  | AIRV 20% | 1.001 | 0.970 | 0.940 - 1.060 | 0.518 |
|  | AIRV 30% | 0.996 | 0.908 | 0.930 - 1.070 | 0.498 |
|  | AIRV 40% | 0.988 | 0.787 | 0.900 - 1.080 | 0.517 |
|  | AIRV 50% | 0.978 | 0.696 | 0.880 - 1.090 | 0.535 |
|  | AIRV 60% | 0.968 | 0.646 | 0.840 - 1.110 | 0.540 |
|  | AIRV 70% | 0.961 | 0.652 | 0.810 - 1.140 | 0.540 |
|  | AIRV 80% | 0.966 | 0.750 | 0.780 - 1.190 | 0.528 |
|  | AIRV 90% | 0.950 | 0.705 | 0.730 - 1.240 | 0.545 |
|  | Absolute Volume of Relative Intensity (AVRI)  (9/9) | | | | |
|  | AVRI 10% | 1.003 | 0.133 | 1.000 - 1.010 | 0.554 |
|  | AVRI 20% | 1.002 | 0.398 | 1.000 - 1.010 | 0.545 |
|  | AVRI 30% | 1.001 | 0.723 | 1.000 - 1.010 | 0.513 |
|  | AVRI 40% | 0.999 | 0.787 | 0.990 - 1.010 | 0.512 |
|  | AVRI 50% | 0.993 | 0.327 | 0.980 - 1.010 | 0.523 |
|  | AVRI 60% | 0.983 | 0.168 | 0.960 - 1.010 | 0.527 |
|  | AVRI 70% | 0.965 | 0.157 | 0.920 - 1.010 | 0.513 |
|  | AVRI 80% | 0.938 | 0.226 | 0.850 - 1.040 | 0.486 |
|  | AVRI 90% | 0.893 | 0.625 | 0.570 - 1.400 | 0.413 |
|  | Mean Intensity of Relative Volume (MIRV)  (9/9) | | | | |
|  | MIRV 10% | 1.016 | 0.506 | 0.970 - 1.060 | 0.568 |
|  | MIRV 20% | 1.012 | 0.657 | 0.960 - 1.060 | 0.557 |
|  | MIRV 30% | 1.009 | 0.758 | 0.950 - 1.070 | 0.546 |
|  | MIRV 40% | 1.006 | 0.839 | 0.950 - 1.070 | 0.536 |
|  | MIRV 50% | 1.004 | 0.896 | 0.940 - 1.070 | 0.527 |
|  | MIRV 60% | 1.003 | 0.941 | 0.930 - 1.080 | 0.513 |
|  | MIRV 70% | 1.001 | 0.972 | 0.930 - 1.080 | 0.512 |
|  | MIRV 80% | 1.000 | 0.992 | 0.920 - 1.090 | 0.509 |
|  | MIRV 90% | 1.000 | 0.996 | 0.910 - 1.100 | 0.493 |
|  | Total Lesion Glycolysis of Relative Intensity (TLGRI)  (9/9) | | | | |
|  | TLGRI 10% | 1.000 | 0.234 | 0.999 - 1.000 | 0.550 |
|  | TLGRI 20% | 1.000 | 0.362 | 0.999 - 1.000 | 0.534 |
|  | TLGRI 30% | 1.000 | 0.565 | 0.999 - 1.000 | 0.527 |
|  | TLGRI 40% | 1.000 | 0.967 | 0.999 - 1.000 | 0.525 |
|  | TLGRI 50% | 1.000 | 0.524 | 0.999 - 1.000 | 0.490 |
|  | TLGRI 60% | 0.999 | 0.268 | 0.999 - 1.000 | 0.498 |
|  | TLGRI 70% | 0.998 | 0.239 | 0.999 - 1.000 | 0.485 |
|  | TLGRI 80% | 0.997 | 0.366 | 0.999 - 1.000 | 0.460 |
|  | TLGRI 90% | 1.002 | 0.862 | 0.980 - 1.030 | 0.587 |

Table 4 - Univariable Cox regression on the robust and stable features of the metastatic lymph nodes from the validation dataset (n=50). Analysis was conducted for features with an ICC(1,k) over 0.85 and within a ±10% LoA interval as derived from the largest (LN_volume_) and more active node (LN_max_) and merged structure (LN_merged_), for the different groups of features, with exception of the ones indicated by an asterisk (*). Volume corresponds to the total volume of all metastatic lymph nodes (†).

| Class of feature | | Feature | | Hazard Ratio | | p-value | | 95% Confidence Interval | | C-index | |
| --- | --- | --- | --- | --- | --- | --- | --- | --- | --- | --- | --- |
| Shape and size (1/13) | Volume ^†^ | | 1.010 | | 0.315 | | 0.991 - 1.029 | | 0.593 | |  |
|  | Surface/Volume | | 0.849 | | 0.328 | | 0.610 - 1.180 | | 0.550 | |  |
| First Order Statistics  (2/16) | Entropy | | 1.364 | | 0.061 | | .986 - 1.887 | | 0.593 | |  |
|  | Maximum* | | 1.064 | | 0.023 | | 1.009 - 1.122 | | 0.595 | |  |
|  | Mean* | | 1.135 | | 0.153 | | 0.954 - 1.349 | | 0.572 | |  |
|  | Peak SUV* | | 1.077 | | 0.034 | | 1.005 - 1.153 | | 0.606 | |  |
|  | Uniformity | | 0.090 | | 0.081 | | 0.006 - 1.348 | | 0.579 | |  |
| Texture  (6/44) | Grey Level Run-length (GLRLM) | | | | | | | | | |  |
|  | Grey Level Non-uniformity | | 1.000 | | 0.949 | | 0.989 - 1.011 | | 0.568 | |  |
|  | Long Run Emphasis | | 0.844 | | 0.761 | | 0.282 - 2.527 | | 0.537 | |  |
|  | Short Run Emphasis | | 5.018 | | 0.656 | | 0.004 - 6053 | | 0.541 | |  |
|  | Short Run High Grey Level Emphasis | | 1.076 | | 0.107 | | 0.984 - 1.176 | | 0.572 | |  |
|  | Grey Level Co-occurrence (GLCM) | | | | | | | | | |  |
|  | Entropy | | 1.248 | | 0.071 | | 0.981 - 1.588 | | 0.598 | |  |
|  | Sum of Average | | 1.031 | | 0.109 | | 0.993 - 1.070 | | 0.570 | |  |
| IVH  (3/45) | Absolute Volume of Relative Intensity (AVRI) | | | | | | | | | |  |
|  | AVRI 90% | | 0.937 | | 0.896 | | 0.355 - 2.473 | | 0.425 | |  |
|  | Mean Intensity of Relative Volume (MIRV) | | | | | | | | | |  |
|  | MIRV 10% | | 1.078 | | 0.044 | | 1.002 - 1.16 | | 0.582 | |  |
|  | MIRV 20% | | 1.085 | | 0.060 | | 0.997 - 1.181 | | 0.580 | |  |
